# O-glycosylation in Cancer: Emerging Paradigms and Prospects for Precision Oncology

**DOI:** 10.7150/ijbs.126296

**Published:** 2026-01-22

**Authors:** Junhao Wei, Shengbao Hu, Wanfang Chen, Hye Song Paek, Guohong Liu, Yunbao Pan

**Affiliations:** 1 Department of Laboratory Medicine, Jiayu Hospital, Zhongnan Hospital of Wuhan University (People's Hospital of Jiayu County), Xianning 437200, Hubei, China.; 2 Department of Laboratory Medicine, Zhongnan Hospital of Wuhan University, Wuhan 430071, Hubei, China.; 3 Department of Laboratory Medicine, Huanggang Central Hospital, Huanggang 438000, China.; 4 Department of Radiology, Zhongnan Hospital of Wuhan University, Wuhan University, Wuhan 430071, China.; 5 Hubei Provincial Clinical Research Center for Molecular Diagnostics, Wuhan 430071, Hubei, China.

**Keywords:** o-glycosylation, protein modification, gene modification, tumor microenvironment, biomarkers

## Abstract

O-glycosylation is a key post-translational modification that profoundly shapes tumor biology by regulating cell proliferation, metastasis, and immune evasion. Aberrant O-glycosylation features truncated glycans such as Tn and sialyl-Tn antigens together with dysregulated glycosyltransferases and promotes oncogenesis in diverse malignancies. This review summarizes recent progress in elucidating the role of O-glycosylation in cancer with emphasis on its effects on cell-surface glycoproteins, intracellular signaling pathways, and emerging RNA modifications. Integration of multi-omics data and machine learning has transformed tumor classification and prognosis prediction through distinct glycosylation signatures and now supports personalized treatment strategies. Newly discovered O-glycosylation of RNA reveals additional regulatory layers and broadens the field of glycosylation research. Targeted interventions including glycosyltransferase inhibitors, gene editing, and combination with immunotherapy demonstrate promising therapeutic potential. Advanced high-throughput tools especially mass spectrometry and enzymatic release methods accelerate biomarker discovery and target validation. Collectively, this review underscores the multifaceted impact of O-glycosylation on cancer progression and treatment response while highlighting the urgent need for continued interdisciplinary collaboration to translate these findings into precision oncology and better patient outcomes.

## 1. Introduction

O-glycosylation represents a major post-translational modification of proteins and regulates diverse biological processes including cell signaling, adhesion, and immune responses. In cancer, aberrant O-glycosylation drives malignant transformation and profoundly affects tumor progression, metastasis, and immune evasion^1^. Dysregulated O-glycans alter protein stability, subcellular localization, and intermolecular interactions, thereby reshaping the functional proteome of cancer cells^1^. The inherent complexity of O-glycosylation, encompassing site-specific alterations and the orchestration by various glycosyltransferases^2^, demands deeper mechanistic insight into its oncogenic functions.

In neoplastic cells, disrupted O-glycosylation triggers profound phenotypic reprogramming, promoting uncontrolled proliferation, epithelial-mesenchymal transition (EMT), invasiveness, and suppression of antitumor immunity 1, 3. These O-glycans activate key oncogenic pathways such as Wnt/β-catenin and phosphatidylinositol 3-kinase/protein kinase B (PI3K/AKT), thereby enhancing metastatic potential and therapeutic resistance^4^-^6^.

Recent advances in glycoproteomics, driven by high-resolution mass spectrometry, chemoenzymatic enrichment strategies, and multi-omics integration, have transformed our ability to interrogate site-specific O-glycosylation landscapes with unprecedented depth. These technologies have unveiled cancer-specific glyco-signatures and identified key enzymes, such as polypeptide N-acetylgalactosaminyltransferase 1 (GALNT1), as critical drivers of malignant glyco-phenotypes, highlighting the therapeutic promise of targeting glycosylation machinery to suppress tumor growth and dissemination^7^.

O-glycosylation interacts dynamically with other post-translational modifications, especially phosphorylation. These modifications often compete for the same or adjacent serine (Ser)/threonine (Thr) residues and generate complex regulatory networks that control protein function and cell fate in cancer^8^. This review integrates the latest findings on cancer-associated protein O-glycosylation, focusing on molecular mechanisms, functional impact, immune modulation, and clinical translation. By combining protein-centric and genomic views, we aim to establish a solid framework to accelerate glycosylation-based precision oncology strategies.

## 2. Biosynthesis and Structural Types of O-glycosylation

### 2.1 Basic Concepts and Classification of O-glycosylation

**Figure 1:**
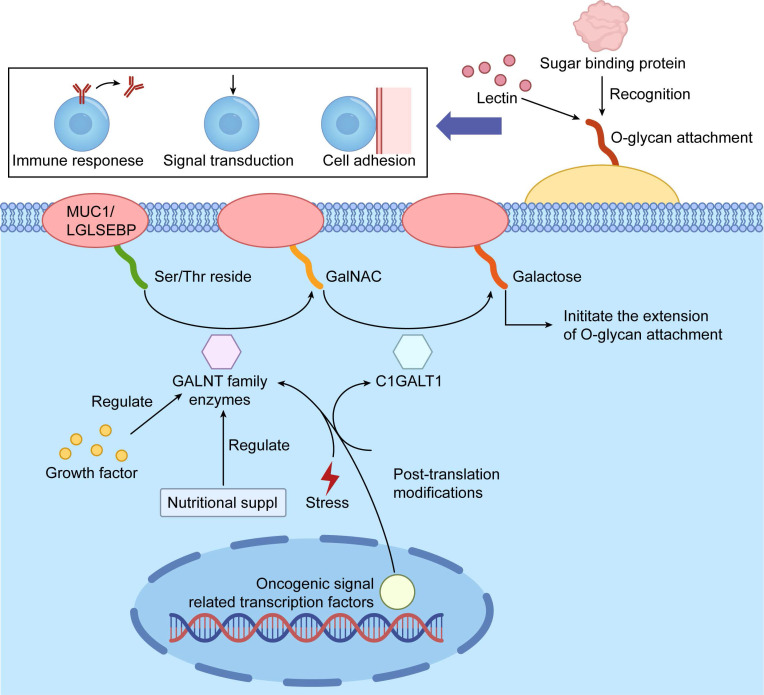
Schematic Illustration of Protein O-glycosylation Initiation and Biological Functions. The figure outlines the key initiation steps of protein O-glycosylation and its critical roles in cellular processes. Specific GALNT family enzymes (N-acetylgalactosaminyltransferases) catalyze the attachment of N-acetylgalactosamine (GalNAc) to Ser or Thr residues, initiating O-glycan chain elongation. This process is regulated by factors such as nutrient availability and cellular stress and can be modulated by other post-translational modifications. The resulting O-glycan structures are recognized by lectins and other glycan-binding proteins, regulating essential processes like cell adhesion, signal transduction, and immune responses, which are pivotal in tumor development and progression.

O-glycosylation represents a pivotal post-translational modification (PTM) characterized by the enzymatic attachment of carbohydrate moieties to the hydroxyl groups of Ser or Thr residues in proteins, facilitated by specialized glycosyltransferases^9^. The inherent complexity of O-glycosylation stems from its lack of consensus sequence motifs and the labile nature of its glycan cores, which hinders site identification. This modification is integral to numerous biological processes, including cell signaling, protein stability, and intercellular interactions^10^, ^11^. O-glycosylation is broadly divided into mucin-type (O-GalNAc) and O-GlcNAc subtypes. Mucin-type O-GalNAc glycosylation begins with the addition of N-acetylgalactosamine (GalNAc) to Ser/Thr residues and predominates in secreted and membrane-bound mucins that form protective barriers in epithelial tissues^12^. In cancer, mucin-type O-GalNAcylation holds particular clinical importance. The pathway is initiated by the polypeptide N-acetylgalactosaminyltransferase (GALNT) family, which transfer GalNAc to Ser/Thr, forming the Tn antigen. Subsequent extension to core 1 (T antigen) is catalyzed by Core 1 beta1,3-galactosyltransferase (C1GALT1) in complex with its essential chaperone Cosmc. Loss-of-function mutations or epigenetic silencing of C1GALT1 or Cosmc, or aberrant GALNT expression cause accumulation of truncated structures such as Tn and sialyl-Tn (STn) antigens. These oncofetal markers strongly associate with enhanced proliferation, immune evasion, metastasis, and poor prognosis in most epithelial cancers 3, 13, 14 (Figure 1).

### 2.2 Key Enzymes and Their Regulation

Mucin-type O-glycan biosynthesis depends on a coordinated enzyme network in which GALNT isoforms and C1GALT1 play central roles^13^. C1GALT1, or core 1 β1,3-galactosyltransferase, is indispensable for synthesizing the core 1 motif by transferring galactose to GalNAc, thereby enabling glycan chain elongation. Its functionality relies on the chaperone Cosmc for proper folding, and perturbations in C1GALT1 expression have been linked to aberrant glycosylation in cancers, driving tumor progression^13^. The GALNT family comprises 20 isoforms with distinct yet partially overlapping substrate specificities, peptide preferences, and subcellular localizations. This diversity enables precise spatiotemporal control of glycan initiation^14^.

GALNT expression is dynamically regulated by growth factors, oncogenic pathways, hypoxia, and metabolic stress, resulting in profound remodeling of the glycoproteome. Regulation occurs at transcriptional, post-transcriptional, and post-translational levels. Phosphorylation and auto-O-GlcNAcylation directly modulate enzyme activity and stability^15^. Dysregulated signaling often amplifies specific glycosyltransferases, yielding glycosylation profiles that support tumor proliferation, immune escape, and metastasis^16^, ^17^. For example, GALNT2 overexpression enhances O-glycosylation of growth factor receptors and sustains proliferation within hostile microenvironments 18.

Enzyme competition and cooperation among glycosyltransferases further refine substrate access and final glycan architecture. Alterations in glycosyltransferase levels can generate lectin-recognizable glycans that mediate cell adhesion, signaling, and immunity^13^, ^19^. Truncated or sialylated O-glycans on tumor surfaces mask antigens, impair immune attack, and facilitate metastasis. Consequently, these enzymes emerge as valuable diagnostic markers and therapeutic targets^20^.

### 2.3 Diversity of O-glycosylation and Tumor-Specific Glycans

Truncated O-glycans, particularly the Tn antigen and STn, represent the most clinically relevant aberrations in cancer^21^. These abbreviated structures are dramatically upregulated in cancers, where they correlate with aggressive phenotypes and unfavorable prognoses 22. Functionally, Tn/STn promote oncogenic signaling, inhibit apoptosis (including TRAIL-induced cell death), and drive epithelial-mesenchymal transition 23, 24. Their aberrant patterns also position them as biomarkers for early detection and immunotherapy targets^25^.

Glycan heterogeneity varies widely across tumor cell types and influences signaling, adhesion, and immune interactions^26^. Prostate cancers, for example, have unique glycosyltransferase profiles that yield distinct truncated O-glycans compared to normal prostate tissue^27^. Such differences modulate lectin recognition and immune cell trafficking, thereby shaping pro-tumorigenic microenvironments 28. Comprehensive mapping of O-glycan diversity is therefore essential for precision diagnostics and therapy (Table 1).

In summary, the cancer-associated shift toward truncated and aberrant O-glycans serves as both a hallmark and active driver of malignancy. Deciphering the responsible enzymes and their regulation provides critical opportunities for early detection, accurate prognostication, and development of glycosylation-directed therapeutics.

## 3. O-glycosylated Proteins in Tumors

### 3.1 Abnormal O-glycosylation of Cell Surface Glycoproteins and Tumor Progression

Dysregulated O-glycosylation of cell-surface and secreted glycoproteins is a major driver of tumor progression, primarily through altered cell adhesion, migration, and invasion. Cancer cells frequently display truncated O-glycans that profoundly change glycoprotein function. For instance, aberrant O-glycosylation of LGALS3BP enhances tumor cell binding to extracellular matrix and promotes invasion and metastasis^34^-^36^. Similarly, altered O-glycosylation of cysteine rich with epidermal growth factor (EGF)-like domains 2 (CRELD2) disrupts normal protein interactions and sustains oncogenic signaling^37^. These glycoproteins often bear truncated O-glycans, such as the Tn antigen and its sialylated derivatives, which are prevalent in diverse malignancies and enable immune evasion while exacerbating tumor aggressiveness^43^. Engagement with cognate lectins or receptors further activates proliferative and survival pathways^38^.

**Figure 2:**
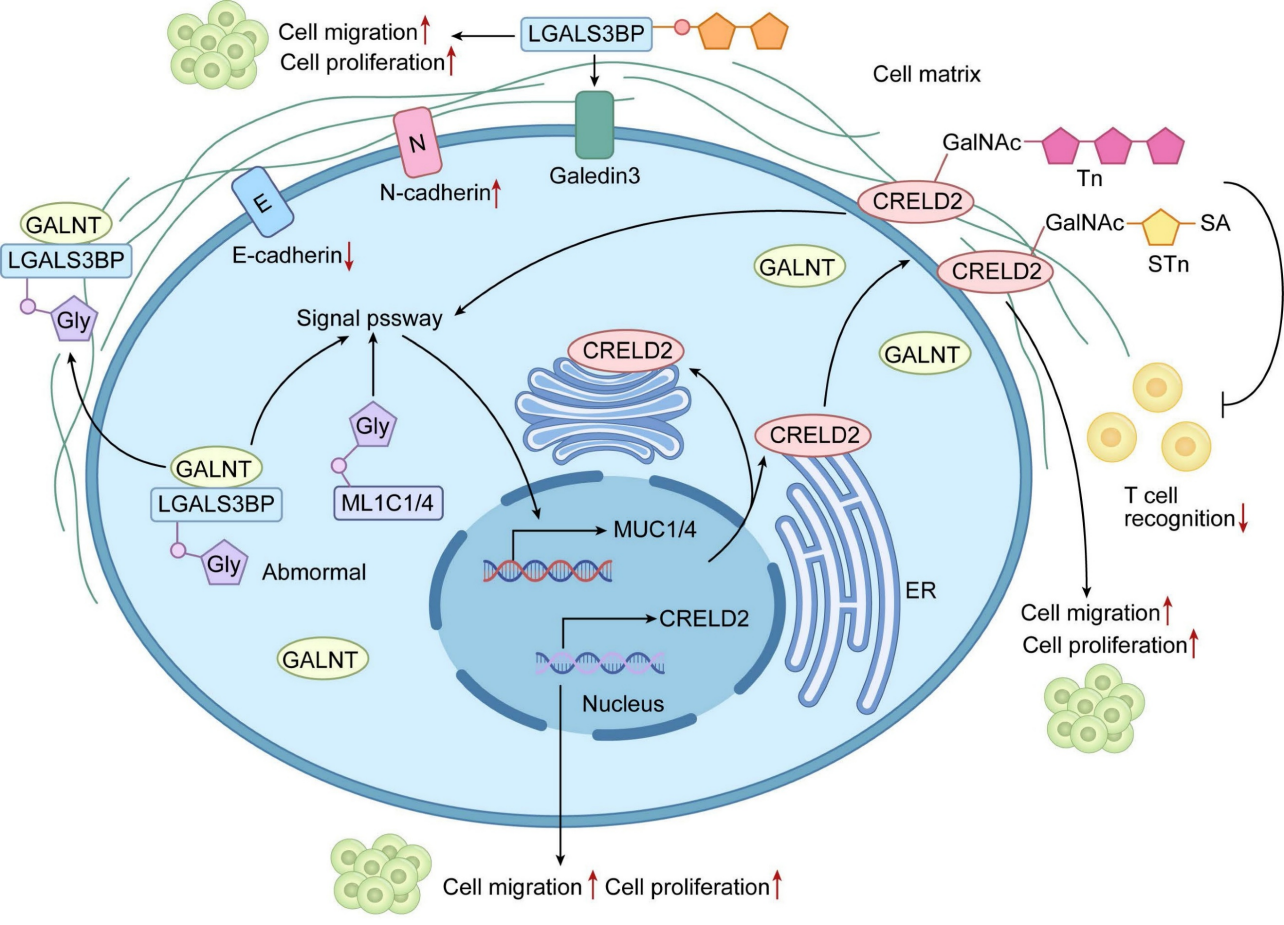
Mechanisms of Aberrant O-glycosylation of Cell Surface Glycoproteins Driving Malignant Phenotypes in Tumors. The diagram elucidates how aberrant O-glycosylation promotes tumor progression by modifying specific cell surface glycoproteins (e.g., LGALS3BP, CRELD2, and MUC1/4). In tumor cells, dysregulated expression or activity of GALNT family enzymes leads to aberrant O-glycosylation. These modifications alter glycoprotein function: LGALS3BP glycosylation enhances tumor cell adhesion to the extracellular matrix, promoting migration and invasion; CRELD2 glycosylation modulates protein interactions, activating oncogenic signaling; and MUC1/4 mucin glycosylation disrupts signaling and adhesion properties. These changes collectively drive core malignant phenotypes, including enhanced proliferation, migration, invasion, and EMT (depicted as E-cadherin to N-cadherin transition), ultimately contributing to tumor progression and metastasis.

Mucins are the most extensively O-glycosylated glycoproteins and play central roles in malignancy. Overexpression and hypoglycosylation of MUC1 and MUC4 create neoepitopes that abolish apical-basolateral polarity, boost proliferation, and confer apoptosis resistance^30^, ^44^, ^45^. Aberrant mucin glycoforms also bind siglecs and galectins on immune and stromal cells, thereby remodeling the tumor microenvironment to favor cancer progression^46^-^48^. Together, these alterations convert cell-surface glycoproteins into active drivers of malignancy, making their modifying enzymes attractive therapeutic targets (Figure 2).

### 3.2 Intracellular O-GlcNAcylation and Oncogenic Signaling

O-GlcNAcylation dynamically modifies nuclear and cytoplasmic proteins and exerts major effects on oncogenic signaling. O-GlcNAcylation of enolase-1 (ENO1) alters its activity and stability, thereby reprogramming glycolytic flux in cancer cells^3^. In ovarian cancer, this modification enhances cell migration and invasion by shifting phosphorylation patterns^49^. Conversely, O-GlcNAcylation of the p53 protein influences its transcriptional efficacy and stability, potentially stabilizing the protein to favor tumorigenesis in specific contexts^49^, ^50^. This bifunctional nature underscores O-GlcNAcylation's dual role in tumorigenesis.

O-GlcNAcylation also functions as a nutrient sensor that integrates glucose, amino acid, and lipid metabolism via the hexosamine biosynthetic pathway (HBP)^51^. Elevated UDP-GlcNAc levels in cancer amplify O-GlcNAcylation of metabolic enzymes and transcription factors 52, 53. This adaptation not only bolsters glycolysis but also aids microenvironmental acclimation, facilitating immune circumvention. O-GlcNAcylation stabilizes PD-L1 through three mechanisms:(1) enhanced transcription via modified STAT3 and NF-κB^54^-^56^, (2) direct modification that blocks ubiquitination and proteasomal degradation^56^, ^57^, and (3) reduced endocytic recycling and lysosomal turnover, resulting in sustained surface expression^58^, ^59^. These effects make the HBP-OGT axis a central amplifier of PD-1/PD-L1 signaling.

In the immune compartment, aberrant O-GlcNAcylation in tumor-associated macrophages drives M2 polarization and immunosuppressive metabolism^60^, ^61^. Inhibition of OGT or the HBP therefore destabilizes oncogenic proteins and PD-L1 in tumor cells while simultaneously restoring antitumor immunity, providing strong rationale for combination with PD-1/PD-L1 blockade^62^, ^63^.

### 3.3 O-glycosylation and the Tumor Immune Microenvironment

**Figure 3:**
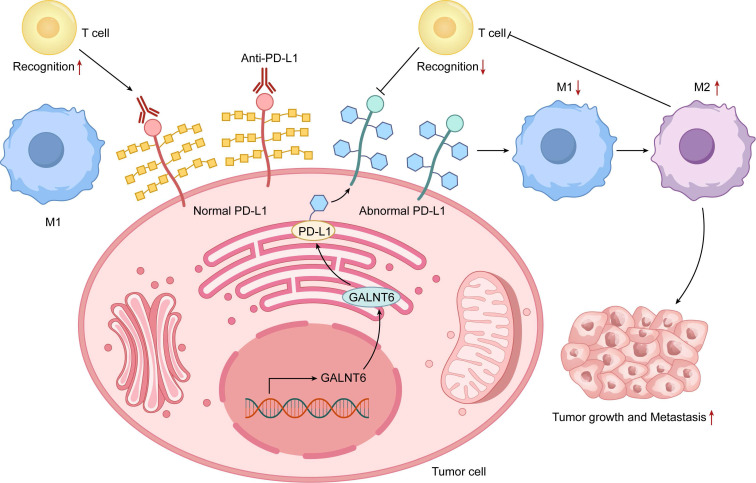
Interactive Diagram of O-glycosylation Regulating the Tumor Immune Microenvironment. The diagram illustrates the dynamic interplay between tumor cells with aberrant O-glycosylation and key immune microenvironment components (T cells and macrophages). The left panel depicts normal immune recognition: unmodified PD-L1 is effectively recognized by T cells, and anti-PD-L1 antibodies function successfully, with macrophages maintaining an antitumor M1 phenotype. The right panel shows an immunosuppressive microenvironment driven by aberrant O-glycosylation (via enzymes like GALNT6): glycosylated PD-L1 on tumor cells impairs T-cell recognition and may reduce immune checkpoint inhibitor efficacy, while glycosylation signals promote macrophage polarization to a protumor M2 phenotype. These mechanisms synergistically drive immune evasion, tumor growth, and metastasis, highlighting O-glycosylation's central role in tumor-immune interactions.

Aberrant O-glycosylation reshapes tumor-immune crosstalk within the tumor immune microenvironment (TIME)(Figure 3). Aberrant modifications on tumor cells influence engagements with macrophages and cytotoxic T lymphocytes (CTLs)^6^, ^64^. Tn antigen expression, for instance, polarizes macrophages toward an M2 phenotype and suppresses antitumor immunity^65^. These glycan patterns also impair CTL recognition and killing, contributing to primary or acquired resistance to checkpoint inhibitors^66^, ^67^.

Combining glycosylation inhibitors with immune checkpoint blockade emerges as a powerful synergistic strategy^68^. Inhibition of GALNT6 in pancreatic cancer increases cytotoxic T-cell and macrophage infiltration and enhances immune attack^66^. Such approaches simultaneously restore immune surveillance and directly target tumor cells^69^. As understanding of glycosylation-mediated immune modulation deepens, targeting aberrant O-glycans is gaining traction as a new pillar of cancer immunotherapy (Table 2).

## 4. O-glycosylation-Related Genes and Their Expression and Function in Tumors

### 4.1 Expression Profiles and Regulation of O-glycosylation-Related Genes

**Figure 4:**
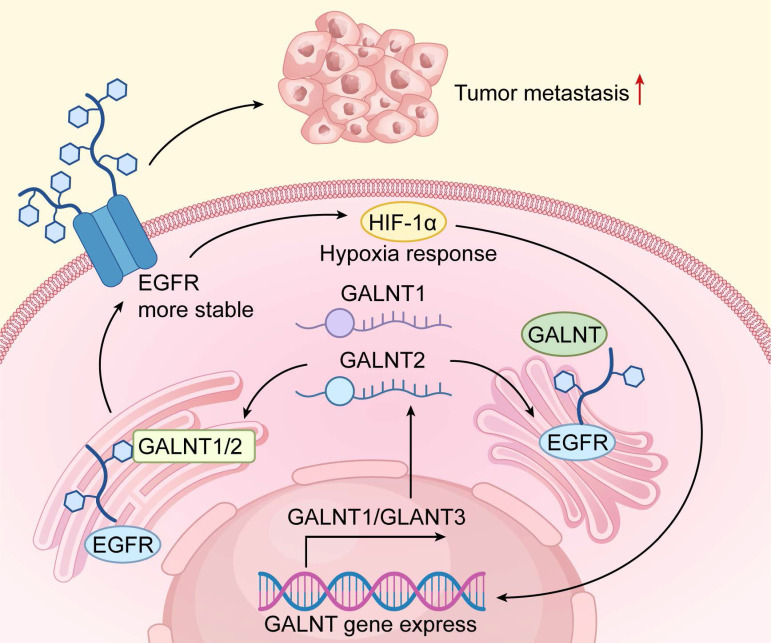
Functional Mechanisms of GALNT Family Genes in Tumor Progression. The mechanistic diagram delineates the central role of GALNT family genes in tumor progression. Under tumor microenvironment stimuli (e.g., hypoxia), transcription factor HIF-1α is activated, upregulating GALNT1 and other GALNT genes. These genes encode GALNTs that catalyze O-glycosylation of downstream substrates (e.g.,EGFR). This post-translational modification enhances receptor stability, sustaining activation of intracellular signaling pathways that promote tumor cell proliferation, survival, and metastasis. The diagram clearly maps the signaling axis from microenvironmental cues to gene expression, protein modification, and malignant phenotype manifestation.

Recent studies reveal widespread dysregulation of O-glycosylation genes across tumor types and highlight their central role in cancer biology^75^-^77^. In hepatocellular carcinoma (HCC), transcriptomic analyses show striking changes in GALNT family members compared with adjacent normal liver. These expression patterns faithfully mirror molecular HCC subtypes and strongly predict clinical outcome^77^. Similar upregulation of C1GALT1 and specific GALNTs has been documented in colorectal and breast carcinomas, where high expression correlates with aggressive behavior, immune exclusion, and poor prognosis^78^-^80^. These findings underscore the need for detailed mechanistic studies of glyco-gene regulation (Figure 4).

Transcriptional control is tightly coupled to the tumor microenvironment. Hypoxia-inducible factor-1α (HIF-1α), activated in hypoxic niches, directly transactivates several GALNTs, driving the synthesis of truncated O-glycans that facilitates metastasis^81^. Extensive crosstalk with Wnt/β-catenin, MYC, and other oncogenic pathways further amplifies this response^82^. Dysregulated O-glycosylation genes and their upstream regulators thus represent promising therapeutic targets to improve treatment efficacy (Table 3).

### 4.2 O-glycosylation Genes in Molecular Subtyping of Tumors

Integration of O-glycosylation gene expression with machine learning has transformed tumor classification, especially in HCC. Consensus clustering using GALNT1, GALNT2, GALNT6, and related genes defines robust molecular subclasses that differ in proliferation rate, immune infiltration, and survival^83^, ^87^, ^88^. GALNT1 and GALNT2 directly modulate EGFR O-glycosylation and downstream signaling strength, providing a clear mechanistic link to oncogenic driver activity^83^.

Single-cell analyses further resolve intratumoral heterogeneity and predict treatment response. High GALNT6 expression, for example, strongly correlates with lenvatinib resistance in HCC, while distinct glyco-subtypes show variable sensitivity to immune checkpoint blockade^84^. These results position O-glycosylation gene panels as clinically valuable tools for precision oncology and patient stratification.

### 4.3 Mutations in O-Glycosylation Genes and Tumorigenesis

Somatic mutations and epigenetic silencing of core O-glycosylation genes, most notably Core 1 beta 3-Gal-T-Specific Molecular Chaperone (COSMC /C1GALT1C1) and C1GALT1, are recurrent events in cancers and represent a direct genetic mechanism for the exposure of truncated O-glycans^89^. In triple-negative breast cancer (TNBC), COSMC mutations drive Tn antigen overexpression, which not only serves as a diagnostic marker but also actively reshapes the immune microenvironment^41^. Tn-positive tumors recruit immunosuppressive macrophages through galectin and siglec interactions while suppressing cytotoxic T-cell activity 72. These alterations confer enhanced metastatic potential and broad therapeutic resistance. Genetic disruption of O-glycosylation pathways therefore emerges as a fundamental oncogenic driver with major implications for tumor progression and clinical management.

## 5. O-glycosylation of RNA

### 5.1 Discovery and Detection of RNA O-Glycosylation

The discovery of cell-surface glycosylated RNAs (glycoRNAs) in mammalian cells has revealed a novel class of biomolecules that intersects glycobiology and RNA biology^90^. Although their physiological functions remain poorly defined, initial evidence points to potential roles in cell-cell communication and immune recognition.

Technological advances have driven early progress in this emerging field. The Tn-containing O-glycosylated RNAs (TnORNA) method stands out as a pioneering chemoenzymatic approach for capturing and enriching O-glycosylated RNA^86^, ^90^. Using this approach, several miRNAs including miR-103a-3p and miR-122-5p have been identified as O-glycosylated in pancreatic cancer models, with suggested effects on PI3K-Akt signaling and proliferation^86^. Complementary computational tools such as PONglyRNA now predict glycosylation sites on RNA with high confidence and support experimental design^86^, ^91^. Despite these advances, the stoichiometry, tissue distribution, and functional impact of RNA O-glycosylation are still inadequately characterized. Independent replication of initial findings remains essential.

### 5.2 O-glycosylated miRNAs in Tumors

Limited but intriguing data indicate that certain miRNAs undergo direct O-glycosylation, which may influence their stability, localization, or target engagement. In pancreatic ductal adenocarcinoma, TnORNA studies report O-glycan attachment to miR-103a-3p and miR-122-5p. These modifications correlate with increased PI3K-Akt activation and enhanced proliferation 86. Similarly, in HCC, dysregulation of the miR-424-5p/OGT axis has been implicated in oncogenic networks, though direct RNA glycosylation in this context requires verification 39.

Current evidence is primarily descriptive. The consequences of O-glycan addition to miRNAs—such as protection from degradation, altered argonaute binding, or extracellular interactions—remain unproven. The enzymes that catalyze RNA O-glycosylation or remove these marks are still unknown. Thus, while O-glycosylated miRNAs represent an exciting concept, the field is in its infancy. Assertions of clear oncogenic functions should await rigorous mechanistic validation.

### 5.3 RNA O-glycosylation and Tumor Signaling Pathways

**Figure 5:**
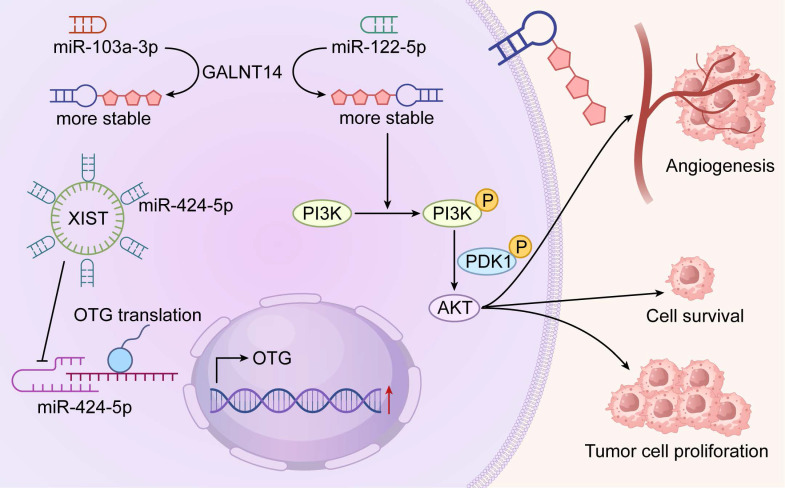
Mechanisms of RNA O-glycosylation in Promoting Tumor Progression through the PI3K-Akt Pathway. Schematic illustration depicting the molecular mechanisms by which RNA O-glycosylation drives tumor progression via sustained activation of the PI3K-Akt pathway. In tumor cells, the long non-coding RNA XIST modulates miR-424-5p expression, which in turn regulates the activity of key glycosylation enzymes, including O-GlcNAc transferase (OGT). Concurrently, O-glycosylation of specific miRNAs (e.g., miR-103a-3p and miR-122-5p, markedly enhancing their stability and prolonging their functional lifespan. These glycosylated miRNAs promote persistent PI3K/AKT pathway activation by suppressing tumor suppressor genes and/or negative regulators. The resulting hyperactivation of the PI3K-Akt signaling cascade ultimately orchestrates multiple pro-tumorigenic phenotypes, including enhanced angiogenesis, increased cell survival, and accelerated tumor cell proliferation, collectively driving tumor progression and metastatic dissemination.

RNA O-glycosylation emerges as a new frontier in cancer biology with potential links to key signaling pathways such as PI3K-Akt (Figure 5). Reported glycosylation of miR-103a-3p and miR-122-5p in pancreatic and lung adenocarcinoma correlates with sustained pathway activity and chemoresistance 40, 86. In lung cancer, GALNT14-driven protein O-glycosylation similarly boosts proliferation, prompting questions about coordinated regulation of protein and RNA glycosylation 20. By contrast, GALNT8-mediated glycosylation in breast cancer suppresses metastasis by dampening EGFR signaling and highlights context-specific effects^85^.

Computational tools now facilitate transcriptome-wide prediction of glycosylation sites and guide targeted investigations^86^. However, causal evidence connecting RNA O-glycosylation to altered signaling remains limited. The field requires prioritized efforts in independent validation, enzyme identification, and precise functional studies to distinguish correlation from true regulatory mechanisms.

## 6. O-glycosylation as a Target for Tumor Diagnosis and Therapy

### 6.1 O-glycosylation-Related Biomarkers

Tumor-associated carbohydrate antigens (TACAs) generated by aberrant O-glycosylation, including Tn, STn, and CA19-9, serve as established diagnostic and prognostic markers. Tn antigen is overexpressed in many adenocarcinomas and detectable in tissue biopsies and serum, allowing non-invasive monitoring of tumor burden and therapy response. STn similarly predicts aggressive disease and poor outcome, while CA19-9 remains the standard serum marker for pancreatic cancer 42, 92. Salivary glycoproteomics has identified distinct sialylation and fucosylation patterns in early lung cancer, supporting non-invasive screening approaches^93^, ^94^. High-resolution mass spectrometry platforms now enable site-specific and quantitative glycan analysis, driving discovery of more sensitive and specific biomarkers^95^, ^96^ (Table 4).

Clinical translation of O-glycosylation biomarkers faces substantial challenges. Glycosylation patterns exhibit profound heterogeneity at multiple levels. Inter-patient variation arises from genetics, microbiome, and metabolism^100^. Intra-tumor heterogeneity, driven by subclonal diversity and microenvironmental gradients such as hypoxia, creates spatially variable glyco-profiles^28^, ^101^. This mosaicism causes sampling bias in biopsies and dilutes tumor signals in liquid biopsies. Truncated glycans like Tn also appear in benign inflammation, reducing specificity^102^. Technical hurdles include O-glycan lability, low site occupancy, scarcity of reliable site-specific antibodies, and limited clinical compatibility of glycoproteomics workflows.

### 6.2 Therapeutic Targeting of O-glycosylation

Pharmacological inhibition of O-glycosylation emerges as a promising adjuvant to immunotherapy. Repurposed itraconazole suppresses glycosylation in head and neck cancer and markedly improves anti-PD-1 response by shifting macrophages to an M1 phenotype, enhancing CD8+ T-cell activity, and lowering immunosuppressive cytokines^6^. These benefits arise from remodeled tumor-surface glycans that disrupt inhibitory immune checkpoints.

**Figure 6:**
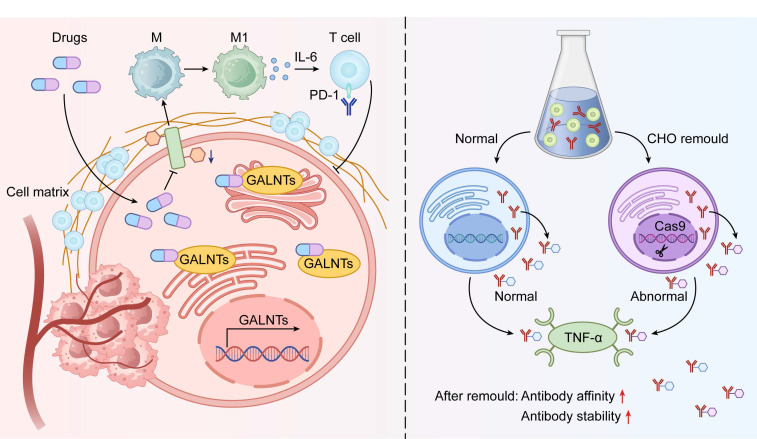
Schematic of Therapeutic Strategies Targeting O-glycosylation. The diagram integrates two key therapeutic strategies targeting tumor O-glycosylation. The left panel depicts small molecule inhibitors that suppress GALNT enzyme activity, blocking aberrant O-glycosylation and reversing immunosuppressive tumor microenvironments. This approach synergy with immune checkpoint inhibitors. The right panel showcases glycosylation engineering platforms, using CRISPR/Cas9-modified CHO cells to optimize therapeutic proteins (e.g., antibodies or receptor fusion proteins) by tailoring O-glycosylation, improving binding affinity and stability to targets like TNF-α. These strategies collectively highlight the multidimensional therapeutic potential of targeting O-glycosylation from microenvironment modulation to biotherapeutic optimization.

Gene editing and enzyme modulation offer additional avenues. CRISPR/Cas9 knockout of C1GALT1 induces Tn/STn exposure, disrupts oncogenic signaling, and sensitizes colorectal and pancreatic tumors to immune clearance^98^. Selective glycosyltransferase inhibitors alter tumor antigen glycans to boost immune recognition^103^ (Figure 6). These strategies simultaneously impair tumor cell fitness and overcome immune evasion. Continued development of O-glycosylation-targeted therapies holds potential for personalized treatment across cancer types (Table 5).

### 6.3 O-glycosylation Engineering Cell Platforms and Biotherapeutic Optimization

Glycoengineering of O-glycosylation in producer cells has become essential for optimizing biopharmaceuticals. Specialized Chinese hamster ovary (CHO) platforms now allow precise control of O-glycan structures on recombinant proteins^106^. These systems produce variants with defined glycans that influence activity and pharmacokinetics. For example, etanercept analogs bearing truncated (Tn/STn) or extended (sialyl-core 1/3) O-glycans show improved TNF-α binding and therapeutic efficacy^99^. Such findings establish O-glycosylation as a critical quality attribute in biotherapeutic development.

Selective O-glycan modulation preserves N-glycosylation and overall protein integrity^13^. This approach enhances performance while enabling systematic study of glycan effects on product quality. Broader application will improve diverse biologics and deliver superior patient outcomes. Robust glycoengineered platforms are therefore vital for advancing biopharmaceutical manufacturing to modern standards.

## 7. Technological Advances and Future Research Directions

### 7.1 Development of High-Throughput Analysis Technologies

High-throughput technologies have revolutionized glycosylation research in oncology. Ultrahigh-resolution mass spectrometry, including MALDI-FT-ICR MS, enables simultaneous profiling of N- and O-glycans. These methods provide detailed structural analysis of complex glycans from cancer cell lines and tissues. A semi-automated workflow has profiled O-glycans in colorectal and pancreatic cancer cells, identifying compositions from monosaccharides to branched oligosaccharides^97^. Automated tools like MassyTools improve quantification accuracy using signal-to-noise and mass error criteria across large datasets^97^.

Glycopeptide enrichment strategies have further advanced O-glycosylation analysis. The MOTAI method combines enzymatic digestion with solid-phase extraction to boost sensitivity and coverage in tumor samples^37^. This approach has identified upregulated Tn/STn-glycoproteins in colorectal cancer compared with normal tissue, highlighting diagnostic potential. Sequential O-glycoprotease digestion in MOTAI isolates O-GalNAc sites and elucidates aberrant glycoprotein functions 37.

Integration with high-throughput sequencing and bioinformatics correlates glycan profiles with clinical outcomes 107. Ongoing refinements will deepen insights into glycosylation-cancer interactions and refine diagnostic paradigms^107^, ^108^.

### 7.2 Multi-omics and Machine Learning Applications in O-glycosylation

Multi-omics integration elucidates O-glycosylation networks in tumors. Weighted gene co-expression network analysis (WGCNA) in HCC identifies key glyco-genes and defines molecular subtypes linked to behavior and prognosis^77^. Subtype CS1 shows genomic heterogeneity and moderate immune infiltration. Subtype CS2 exhibits genomic stability and favorable immune profiles 77. Combining glycomics with genomics and transcriptomics reveals O-glycosylation effects on the tumor microenvironment, immune phenotypes, and outcomes, aiding biomarker and target discovery 77, 109.

Machine learning enhances O-glycosylation-based classification and prognostication^110^. Evaluation of 59 algorithms has yielded robust HCC prognostic models from glyco-gene expression, defining clinically relevant subtypes^77^. CS2 patients display superior survival and strong immune infiltration, predicting immunotherapy response. CS3 patients face poor prognosis with genomic instability, requiring alternative approaches^77^. These machine-learning signatures support clinical decision-making and patient stratification. Multi-omics and machine learning thus drive personalized oncology for HCC and beyond^77^.

### 7.3 New Opportunities for O-glycosylation Modification in Tumor Immunotherapy

O-glycosylation regulates tumor immunobiology by modulating immune checkpoints and cell function. Aberrant O-glycans on tumor glycoproteins stabilize PD-L1, impair T-cell activation, and promote immune evasion 56. Targeting these modifications may boost checkpoint inhibitor efficacy. Glycopeptide epitopes from CD44v6 have enabled development of selective monoclonal antibodies with enhanced tumor targeting and safety^74^, ^111^. Personalized glycopeptide vaccines mimicking TACAs could elicit strong antitumor immunity and overcome resistance^104^.

Sialylated O-glycans form a protective glycocalyx that shields tumors from immune attack^112^. Inhibitors or glycan-engineered immune cells that disrupt sialylation may synergize with immunotherapies^113^. Glycosylation profiles also predict immunotherapy response and guide treatment selection 114, 115. The immunomodulatory roles of O-glycosylation merit intensive study to advance cancer immunotherapy and patient outcomes.

### 7.4 Future Exploration in RNA Glycosylation

RNA glycosylation represents a new regulatory layer with major implications for cancer. GlycoRNAs on small RNAs modulate immune interactions and tumor progression^90^, ^116^, ^117^. Aberrant glycoRNA patterns associate with malignancy and resistance^117^, ^118^. Coexistence of N- and O-linked modifications on RNAs suggests interplay with protein glycosylation, influencing gene regulation and signaling^86^. Future studies should define glycoRNA functions and cross-talk with protein pathways to uncover therapeutic avenues.

Advanced detection technologies are essential for progress. Current methods lack sensitivity, necessitating tools like solid-phase chemoenzymatic gRNA (SPCgRNA) for selective capture and profiling 105. Bioinformatics predictors such as PONglyRNA identify RNA glycosylation sites for hypothesis testing^86^. Extension to long non-coding RNAs, circular RNAs, and other classes will clarify roles in disease.

Therapeutic targeting of RNA glycosylation offers novel oncology strategies. Altering glycoRNA pathways may influence RNA stability, cellular localization, and downstream signaling, thereby affecting tumor proliferation, apoptosis, and immune evasion^117^, ^119^. Multi-omics integration will decode the cancer glyco-code and enable personalized approaches. Sustained RNA glycosylation research promises key insights and innovative treatments.

## 8. Conclusion

Glycosylation profoundly influences tumor biology by regulating proliferation, metastasis, and immune interactions. Aberrant forms, characterized by truncated glycans such as Tn and STn antigens alongside dysregulated glycosyltransferases, actively drive oncogenesis and disease progression. Integration of multi-omics data with machine learning has transformed tumor subtyping, prognostic modeling, and treatment selection. These tools uncover cancer-specific glycosylation signatures and enable personalized interventions to improve outcomes.

The discovery of RNA O-glycosylation introduces an exciting new regulatory layer. GlycoRNAs hold promise as diagnostic markers, prognostic indicators, and therapeutic targets, warranting intensive study to integrate them into precision oncology. Therapeutically, targeted modulation of aberrant O-glycosylation, particularly in combination with immunotherapy, yields promising preclinical and early clinical results that may expand treatment efficacy.

Significant barriers persist in clinical translation. The inherent complexity of O-glycans—including low stoichiometry, heterogeneity, and technical detection challenges—necessitates continued advances in mass spectrometry, enrichment strategies, and analytical workflows. Large-scale validation studies and interdisciplinary collaboration among glycobiologists, oncologists, immunologists, bioinformaticians, and clinicians remain crucial. Overcoming these obstacles will unlock the full potential of O-glycosylation research to deliver innovative diagnostic tools and transformative therapies in precision oncology.

## Figures and Tables

**Table 1:** Functions of Aberrant O-glycosylation

O-glycosylation	Associated Molecules/Antigens	Role in Tumor Biology	Related Cancers	References
Tn antigen expression	MUC1, CD44	Promotes EMT, migration, and metastasis	Breast cancer	29, 30
STn antigen expression	TAG-72, MUC16	Enhances immune evasion, suppresses T-cell infiltration	Colorectal cancer, Ovarian cancer	31, 32
O-GlcNAc modification	C-Myc	Regulates the tricarboxylic acid cycle	Colorectal cancer	33
Aberrant O-glycosylation of LGALS3BP	Galectin-3 binding protein (LGALS3BP)	Enhances adhesion to extracellular matrix, promotes invasion and metastasis	Multiple solid tumors	34-36
Aberrant O-glycosylation of CRELD2	CRELD2	Alters protein interactions, activates oncogenic signaling pathways	Various cancers	37
Aberrant mucin glycosylation	MUC1, MUC4	Promotes proliferation, metastasis, and immune evasion; MUC1 linked to apoptosis resistance	Breast, Pancreatic, Ovarian, Colorectal cancers	38
RNA O-glycosylation	O-glycosylated miR-103a-3p, miR-424-5p	Modulates miRNA stability and function; regulates PI3K-Akt signaling; impacts tumor cell growth and metastatic potential	Liver cancer, Lung cancer	39, 40
Truncated O-glycans	Tn antigen, STn antigen	Facilitate immune evasion, contribute to aggressive phenotype	Multiple cancers	24, 41
Over-sialylated O-glycans	MUC1, MUC16	Enhance tumor immune evasion, promote metastasis	Lung cancer	42

**Table 2:** O-glycosylation in Immune Microenvironment

Target/Process	Regulation	Immune Consequence	References
PD-L1 glycosylation	Activation (↑)	Suppresses T-cell activity → Immune evasion	66, 70
GALNT6 activity	Upregulation	Reduces CTL and macrophage infiltration; promotes immunosuppressive TIME	66
Tn antigen expression	Aberrant O-glycosylation	Facilitates immune evasion; promotes tolerogenic microenvironment	71, 72
Macrophage polarization	M2 phenotype (↑)	Pro-tumor inflammation, angiogenesis	65
CTL recognition of tumor cells	Altered by glycosylation	Decreased cytotoxic function	6
Sialic acid-Siglec binding	Enhancement (↑)	Inhibits NK cell cytotoxicity	28, 73
O-glycosylation of tumor cell surface	Aberrant	Modulates interactions with immune cells; promotes immune evasion	74

**Table 3:** Regulatory Function of GALNT Family

Gene/miRNA	Regulatory Mechanism	Downstream Effect	Cancer Type & Prognostic Link	References
GALNT1	CD44 glycoproteins modified with abundant Tn antigens	Activating the Wnt/β-catenin signaling pathway	Gastric cancer; higher GALNT1 expression had poorer OS and disease-free survival	7
GALNT2	Modulates EGFR expression	Promotes cancer cell proliferation and survival; correlates with HCC molecular subtypes	HCC; linked to tumor progression and prognosis	83
GALNT6	Alters O-glycosylation pathways	Lenvatinib resistance; impacts immune cell infiltration and therapy sensitivity	HCC; high expression correlates with drug resistance and treatment stratification	84
C1GALT1	Upregulated in tumors	Facilitates immune evasion and tumor growth; affects immune microenvironment	Prostate and breast cancers; associated with aggressive behavior and Tn-positive tumors	78
COSMC (C1GALT1C1)	Mutation disrupts O-glycosylation	Aberrant Tn antigen expression; promotes tumor growth and immune evasion	Breast cancer; linked to poor prognosis	41
T-synthase (C1GALT1)	Mutation disrupts O-glycosylation	Tn antigen accumulation; altered gene expression promoting metastasis	Breast cancer; associated with aggressive phenotype	80
OGT (O-GlcNAc transferase)	Modulates O-glycosylation of miR-424-5p	Influences expression of oncogenes and tumor suppressor genes	Liver cancer; affects tumor progression	39
GALNT8	Modulates O-glycosylation affecting EGFR signaling	Suppresses metastatic potential	Breast cancer; regulates tumor metastasis	85
GALNT14	Initiates O-GalNAcylation	Upregulates oncogenic factors, promotes proliferation and invasion	Lung adenocarcinoma; promotes tumor growth	20
miR-103a-3p	O-glycosylation captured by TnORNA method	Regulates cancer cell proliferation and metastatic potential via PI3K-Akt pathway	Pancreatic cancer; aberrant expression linked to tumor growth and metastasis	86
miR-122-5p	O-glycosylation captured by TnORNA method	Modulates cancer cell proliferation and survival via PI3K-Akt pathway	Pancreatic cancer; aberrant expression promotes tumor progression	86
miR-424-5p	O-glycosylation modulated through interaction with O-GlcNAc transferase (OGT)	Influences expression of oncogenes and tumor suppressor genes	Liver cancer; affects cancer progression	39

**Table 4:** O-glycosylation-Derived Diagnostic Biomarkers

Biomarker	Sample Type	Clinical Utility	References
Tn/STn antigens	Serum, Tissue	Early screening, prognosis evaluation	71
Sialylated glycoproteins	Saliva	Non-invasive lung cancer screening	42
Glycan profiles detected by mass spectrometry	Body fluids (e.g., saliva, serum)	Detailed characterization of glycosylation changes for novel biomarker identification	97
PD-L1 glycosylation	Tumor tissue	Predictive biomarker for immunotherapy (anti-PD-1/PD-L1 efficacy)	66
M2 macrophage-associated O-glycosylation signatures	Tumor microenvironment	Indicator of immunosuppressive tumor state	65
Sialic acid-Siglec binding patterns	Tumor tissue, immune cells	Biomarker of NK cell suppression and immune escape	28
C1GALT1 downregulation-associated O-glycosylation profile	Colorectal cancer tissue	Associated with tumor aggressiveness; potential prognostic biomarker	98
Etanercept variants with distinct O-glycosylation patterns (sialylTn, Tn, sialylCore 3, sialylCore 1)	Engineered therapeutic protein (CHO platform)	Quality attribute biomarker for therapeutic efficacy	99
O-glycosylation pattern	Tumor tissue	Serves as a predictive biomarker for response to combination therapy with itraconazole and anti-PD-1	6

**Table 5:** Strategies Targeting O-glycosylation

Strategy	Representative Approach	Mechanism of Action	References
Therapeutic Targeting of Tumor-Specific Glycopeptides	Development of monoclonal antibodies targeting glycopeptide epitopes	Specific targeting of glycopeptides to enhance tumor cell recognition while minimizing damage to healthy tissue	74
Glycosyltransferase Inhibitors	Itraconazole	Blocks GALNT activity → Inhibits PD-L1 glycosylation	6
Glycoengineering	CHO cell platform	Optimizes O-glycan structures → Improves binding affinity	99
Gene Editing	CRISPR-mediated GALNT14 knockdown	Suppresses RNA O-GalNAc modification → Blocks PI3K pathway	20
Glycosylation-Targeted Cancer Vaccines	Glycopeptide-based vaccines targeting tumor-associated carbohydrate antigens	Induces immune responses against tumor-specific glycosylation markers, aiding in the recognition and destruction of tumor cells	104
Glycosylation inhibitors	Disrupt tumor-associated glycocalyx	Disrupts the protective glycocalyx formed by sialylated O-glycans on tumor cells, enhancing immune recognition	6
RNA Glycosylation	Targeting glycosylated RNA (glycoRNA)	Regulates immune responses and tumor progression	86, 105

## Abbreviations

EMT: Epithelial-mesenchymal transition
PI3K/AKT: Phosphatidylinositol 3-kinase /protein kinase B
GALNT1: Polypeptide N-acetylgalactosaminyl-transferase 1
PTMs: post-translational modifications
Ser: Serine
Thr: Threonine
O-GalNAc: Mucin-type O-linked N-acetylgalactosamine
GalNAc: N-acetylgalactosamine
GALNT: Polypeptide N-acetylgalactosaminyltransferase
C1GALT1: Core 1 beta 1,3-galactosyltransferase
STn: Sialyl Tn antigen
GalNAc-T: N-acetylgalactosaminyltransferase
LGALS3BP: Galectin-3-Binding Protein
CRELD2: cysteine rich with epidermal growth factor (EGF)-like domains 2
MUC1: Mucin 1
ENO1: Elpha-Enolase
HBP: Hexosamine biosynthetic pathway
PD-L1: Programmed cell death ligand 1
STAT3: Signal transducer and activator of transcription 3
NF-kappaB: Nuclear Factor Kappa-light-chain-enhancer of Activated B Cells
PDL-1: Programmed death ligand 1
PD-1: programmed cell death protein-1
OGT: O-GlcNAc transferase
TIME: Tumor immune microenvironment
CTLs: Tytotoxic T lymphocytes
PDAC: Pancreatic Ductal Adenocarcinoma
HCC: Hepatocellular carcinoma
HIF-1α: Hypoxia-inducible factor 1α
EGFR: Epidermal growth factor receptor
COSMC /C1GALT1C1: Core 1 beta 3-Gal-T Specific Molecular Chaperone
TNBC: Triple negative breast cancer
GlycoRNAs: Glycosylated RNAs
TnORNA: Tn-containing O-glycosylated RNAs
PONglyRNA: Predictor of O-and N-linked glycoRNA
EGFR: Epidermal growth factor receptor
TACAs: Tumor-associated carbohydrate antigens
CHO: Chinese hamster ovary
TNF-α: Tumor necrosis factor-alpha
MALDI-FT-ICR: Matrix-assisted laser desorption/ionization Fourier transform ion cyclotron resonance
WGCNA: Weighted gene co-expression network analysis
CS1: cancer subtype 1
CD44v6: Cluster of differentiation 44 variant 6
SPCgRNA: Solid-phase chemoenzymatic gRNA
